# Deep learning for early diagnosis of uveal melanoma: a systematic review and meta-analysis

**DOI:** 10.1007/s12094-025-04187-3

**Published:** 2026-01-30

**Authors:** Francisco Cezar Aquino de Moraes, Gustavo Tadeu Freitas Uchôa Matheus, Ísis Larissa de Brito Dichtl, Michele Kreuz, Emanuele Rocha da Silva, Rommel Mario Rodriguez Burbano

**Affiliations:** 1 Department of Clinical and Toxicological Analyses, University of Sao Paulo, Sao Paulo, Sao Paulo Brazil; 2 Federal University of Triângulo Mineiro, Uberaba, Minas Gerais Brazil; 3 Federal University of Piauí, Teresina, Piauí Brazil; 4 Lutheran University of Brazil, Canoas, Rio Grande Do Sul Brazil; 5 Human Cytogenetics Laboratory, Institute of Biological Sciences, Federal University of Pará, Belém, Pará Brazil

**Keywords:** Artificial intelligence, Deep learning, Meta-analysis, Uveal melanoma

## Abstract

**Background:**

Uveal melanoma (UM) is a rare cancer with an estimated annual incidence of 6 incidences per million people. About half of UM patients develop distant metastases, mainly to the liver. After metastasis, prognosis is poor, with median survival under 1 year and limited treatment options. Thus, earlier diagnostic methods for UM are a critical unmet need. The aim of this study was to evaluate the accuracy (sensitivity, specificity, and combined F1 score) of deep learning algorithms in the differential diagnosis of individuals with uveal melanoma.

**Methods:**

We searched PubMed, Scopus, and Web of Science for studies comparing UM patients to healthy individuals or those with ocular nevi using AI diagnostic tools. AI performance metrics were extracted, with clinical or expert-based assessment as the reference standard. The study adhered to PRISMA guidelines.

**Results:**

Five studies comprising 6388 patients (2981 UM, 2563 nevi, and 844 healthy) were included. The mean age of UM patients ranged from 58 to 63.2 years; for nevi, from 58 to 66 years. Pooled sensitivity was 89.0% (95% CI 88.6–89.5%) with no heterogeneity (*I*^2^ = 0.0%, *p* = 0.9242); individual sensitivities ranged from 82.4 to 100.0%. Pooled specificity was 84.9% (95% CI 73.7–91.9%) with significant heterogeneity (*I*^2^ = 72.3%, *p* = 0.006), ranging from 73.7 to 95.0%. The largest cohort had a specificity of 76.0% (95% CI 75.1–76.9%).

**Conclusions:**

While fundus imaging is widely used in outpatient care, multimodal imaging remains limited to specialized clinics. Developing software to analyze fundus images could improve early, noninvasive UM detection and offer a cost-effective diagnostic tool.

**Supplementary Information:**

The online version contains supplementary material available at 10.1007/s12094-025-04187-3.

## Introduction

Uveal melanoma (UM) is a rare malignancy, with an estimated incidence of 6 cases per million population per year [1, 2]. It is the most common primary intraocular tumor in adults and originates from melanocytes in the choroid, iris, and ciliary body, with posterior (choroidal) lesions being the most prevalent. Approximately 50% of patients diagnosed with UM will develop distant metastases over the course of their disease, most frequently to the liver [3]. Once metastasis occurs, prognosis is poor, with median survival typically less than 1 year, and current treatment options remain limited [4]. Thus, the adoption of tools that enable earlier detection of UM—both within the eye and in hepatic metastases—represents a critical unmet medical need. Differentiation between UM and choroidal nevi—benign melanocytic lesions with similar clinical features—is essential for appropriate management [5, 6]. Misclassification of nevi as malignant may lead to unnecessary interventions, such as radiotherapy or enucleation, whereas failure to recognize UM can result in severe consequences, including irreversible visual loss, increased metastatic spread, and death.

Although histopathological analysis of biopsy specimens remains the diagnostic gold standard for tumors, this approach is largely impractical for ocular lesions owing to the risk of performing unnecessary biopsies on benign neoplasms [7]. Consequently, noninvasive imaging modalities are preferred to preserve ocular structure and integrity [8, 9]. Among these, fundus photography, fluorescein angiography, optical coherence tomography, and ultrasonography are most commonly employed [10]. However, these modalities are highly operator-dependent, and their sensitivity for detecting small melanomas—which may closely resemble benign nevi—is limited [8, 11]. Moreover, many of these imaging techniques are not universally available in all ophthalmology clinics, and definitive diagnosis often requires consultation with an ocular oncologist. As a result, the morphological overlap between uveal melanoma and benign lesions frequently yields inconclusive findings, leading to delays in diagnosis, clinical decision-making, and the therapeutic management of uveal melanoma [11].

In recent years, artificial intelligence has emerged as a promising tool to aid significantly in the interpretation of medical images [10, 12, 13]. Ophthalmic diseases, in particular, represent an attractive domain for deep learning applications due to the growing prevalence of image-based investigations, which enables the automation of diagnostic tasks that previously required expert interpretation. Deep learning is a subset of machine learning that employs neural networks capable of automatically learning and extracting features from large datasets, thereby obviating the need for manual feature engineering [14]. In ophthalmology, deep learning has been applied to optic disk and blood-vessel segmentation, lesion detection, and the prediction and monitoring of disease progression. To expand access and reduce costs, several studies have employed deep learning algorithms to screen fundus photographs, achieving high accuracy in distinguishing active retinoblastoma from normal fundus images or images of stable disease [15, 16].

Deep learning is reshaping diagnostic paradigms in oncology; however, its application to uveal melanoma remains scarcely explored, particularly in multimodal imaging studies aimed at enhancing accuracy. If implemented in clinical practice, the socio-economic impact could be substantial, as the integration of automated image analysis software might yield precise referral recommendations and streamline patient care pathways.

Deep learning is currently updating diagnostic approaches in oncology by offering automated, high-accuracy classification. Despite this significant progress and the recognized limitations of current UM diagnostic modalities, namely their operator dependency and the overlap with benign nevi which leads to diagnosis delays, the application of AI to uveal melanoma remains scarcely explored in the literature, particularly in studies focused on optimizing accuracy using multimodal imaging. The potential socio-economic impact of implementing an automated, high-precision image analysis tool in clinical practice is substantial, offering the promise of precise referral recommendations. Accordingly, this systematic review and meta-analysis is the first, to our knowledge, to specifically and comprehensively clarify the aggregated diagnostic performance, including the accuracy, sensitivity, and specificity, of deep learning models for the detection of uveal melanoma using automated ophthalmic imaging, thereby addressing a critical unmet need in oncology.

Thus, this systematic review and meta-analysis of the literature seeks to clarify the accuracy, sensitivity, and specificity of deep learning models for the detection of uveal melanoma using automated ophthalmic imaging.

## Methods

### Protocol registration and study design

This systematic review and meta-analysis was designed and reported in accordance with the preferred reporting items for systematic reviews and meta-analyses (PRISMA) guidelines [17], with detailed checklists provided in Supplementary Tables 1 and 2. The review protocol was prospectively registered in the international prospective register of systematic reviews (PROSPERO) under the ID: CRD420251019554.

### Eligibility criteria

For inclusion in this meta-analysis, studies were required to meet all of the following criteria: (1) they had to include patients diagnosed with uveal melanoma and compare them to healthy individuals or those with ocular nevus; (2) they needed to employ artificial intelligence methods, specifically deep learning algorithms, as the index test for diagnosing uveal melanoma; and (3) they had to report diagnostic performance metrics of the AI algorithms, using either clinical diagnosis or expert-based visual assessment as the reference standard. Only original research articles published in english in peer-reviewed journals were considered eligible, with no restrictions regarding patient age, disease stage, or publication date. Studies were required to employ any form of deep learning architecture designed for image-based classification. No architecture was excluded. This broad inclusion minimizes selection bias related to model choice. Subgroup analyses, where data permitted, were subsequently planned to explore potential heterogeneity arising from specific image modalities or architectural types.

Studies were excluded if they involved comparisons between uveal melanoma and other ocular or systemic diseases; included overlapping populations; were published solely as conference abstracts, preprints, or letters; presented incomplete data or lacked sufficient information to evaluate diagnostic performance; or did not employ AI-based image analysis as the primary diagnostic method. In addition, studies that did not report sufficient data to allow the extraction or reconstruction of diagnostic accuracy parameters, such as true positives (TP), false positives (FP), true negatives (TN), and false negatives (FN), case reports, technical documents, and studies involving animal models or nonhuman tissues were also excluded.

This meta-analysis was designed to address the following research question: Is deep learning an accurate and effective tool for diagnosing uveal melanoma?

### Literature search

A systematic search of the literature was conducted in May 2025 using three electronic databases: PubMed, Scopus, and Web of Science. The search strategy incorporated combinations of controlled vocabulary and free-text terms related to artificial intelligence and uveal melanoma, including: “artificial intelligence”, “machine learning”, “deep learning”, “deep learning algorithm”, “machine intelligence”, “computer vision systems”, “choroidal nevus”, “pigmented choroidal nevus”, “uveal neoplasms”, “uveal melanoma”, and “intraocular melanoma”. Both automated tools and manual screening were employed to remove duplicate records. Additionally, references of the included articles were screened to identify any relevant studies that may not have appeared in the initial search. Title and abstract screening was performed independently by two reviewers (G.T.F.U.M. and Isis) using Rayyan software. Conflicts during the selection process were resolved through consensus or with the input of a third reviewer (F.C.A.M.). Full details of the search strategy, including Boolean operators for each database, are provided in Supplementary Table 3.

### Data extraction

Data extraction was carried out independently by two reviewers using a standardized data collection template, and all extracted information was verified for accuracy by a second reviewer. The following variables were collected from each eligible study: author, year of publication, country of origin, study design, type of deep learning model employed, and imaging modality used. Additionally, the total number of patients in each diagnostic category—uveal melanoma (UM), choroidal nevus, and healthy individuals—was recorded, along with the reported mean or median age of participants per group, when available.

### Outcomes

The primary outcomes of this meta-analysis were the pooled sensitivity, specificity, and F1-score of deep learning algorithms in distinguishing patients with uveal melanoma from those without the disease (i.e., individuals with choroidal nevi or healthy controls). Sensitivity was defined as the ability of the model to correctly identify true positive cases of uveal melanoma, while specificity reflected the correct identification of true-negative cases. The F1-score, calculated as the harmonic mean of precision and recall, was included as an integrated performance metric, particularly relevant in scenarios with class imbalance or when both false positives and false negatives have clinical implications.

### Statistical analysis

All statistical analyses were performed using RStudio version 4.4.1. A bivariate random-effects model was employed to estimate the pooled sensitivity and specificity of the deep learning models, accounting for between-study variability and assuming potential heterogeneity across studies. Summary receiver-operating characteristic (SROC) curves were generated with 95% confidence intervals (CI) and 95% prediction regions (PR) to visualize overall diagnostic performance and estimate the area under the curve (AUC).

Heterogeneity was assessed using the *I*^2^ statistic, with thresholds of 50 and 75% considered indicative of moderate and high heterogeneity, respectively. The pooled F1-score and other proportions were calculated using a random-effects meta-analysis of proportions, based on the DerSimonian and Laird method. Sensitivity analysis was conducted through a leave-one-out approach, sequentially excluding each study to assess its influence on the overall results.

The threshold effect was evaluated by calculating the Spearman correlation coefficient between the logit of sensitivity and specificity. Potential publication bias was assessed using Deeks’ funnel plot asymmetry test. Meta-analytic computations were conducted using the “mada”, “metafor”, “meta”, "devtools", and “diagmeta” packages in R. A *p* value < 0.05 was considered statistically significant for all analyses, except for Deeks’ test, where a *p* value < 0.10 was used to indicate potential publication bias [18].

### Quality assessment

In this systematic review, two independent reviewers (F.C.A.M. and G.T.F.U.M.) applied the QUADAS-AI tool (quality assessment of diagnostic accuracy studies for artificial intelligence) to evaluate both risk of bias and applicability concerns in AI-based diagnostic accuracy studies. QUADAS-AI extends the original QUADAS-2 framework by incorporating five AI-specific domains: Patient Selection, which examines how participants were enrolled and sampled; input data, which addresses the quality, preprocessing, and representativeness of the data fed into the AI system; the AI Model (index test), encompassing algorithm development (architecture, training, and hyperparameter tuning), internal validation (cross-validation or hold-out testing), and external validation on independent cohorts; reference Standard, which appraises the accuracy and consistency of the ground truth; and flow and timing, which considers the sequence and timing of data collection, AI inference, and reference assessment, as well as the management of missing or excluded cases [19, 20].

Each domain was rated for risk of bias (“low”, “high”, or “unclear”) using predefined signaling questions, and the first three domains were further assessed for their applicability to our review question. Discrepancies in domain ratings were resolved by consensus to ensure a transparent and reproducible appraisal of study quality (Supplementary Table 5).

## Results

### Description of study characteristics

**Fig. 1:**
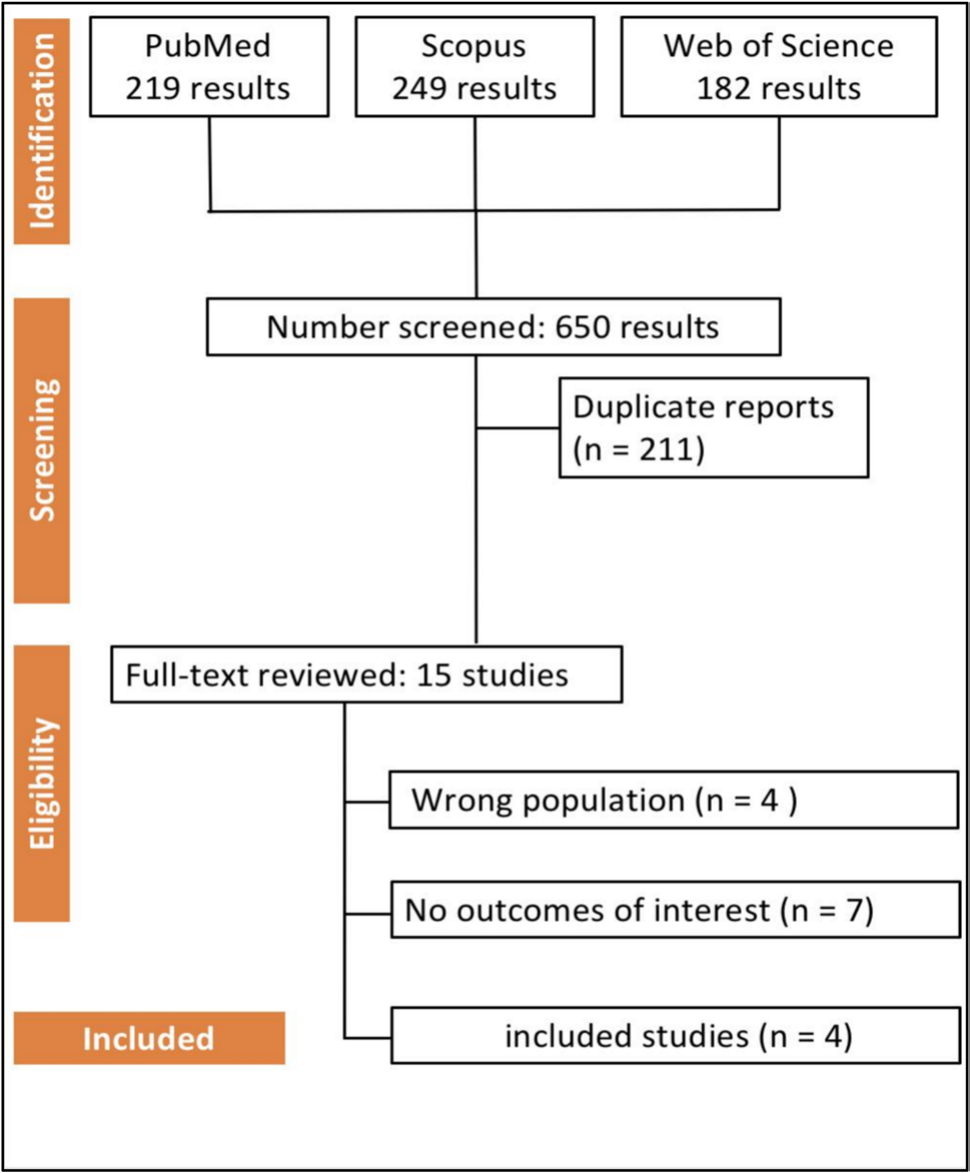
Flow diagram with systematic review and meta-analysis (PRISMA) reporting items. The diagram illustrates the path of information through the different stages of the systematic review

The initial database search yielded 650 records. After duplicate removal, 439 unique studies were screened by title and abstract. Fifteen articles were selected for full-text review, of which 11 were excluded due to irrelevant populations or lack of relevant outcomes. Ultimately, four studies met the inclusion criteria and were included in the quantitative synthesis, as depicted in Fig. 1.

The characteristics of the four included studies are summarized in Table 1. A total of 5950 patients were analyzed, comprising 2824 individuals with uveal melanoma, 2681 with choroidal nevi, and 498 healthy controls. The mean age of patients with melanoma ranged from 58 to 62 years, while those with nevi ranged from 58 to 66 years. Tumor stage and thickness were variably reported: Jackson et al. (2024) [5] provided detailed stratification by stage (T1–T4) and mean tumor thickness (4.0 mm for melanoma, 1.4 mm for nevi), whereas Sabazade et al. (2024) [21] categorized tumors using size thresholds. Regarding diagnostic methodology, all studies utilized automated deep learning models applied to retinal images, including ultra-wide field (UWF) and standard fundus imaging. Model architectures varied, with the use of RETFound, ResNet50, and custom neural networks tailored to the dataset. Diagnostic criteria included MOLES or TFSOM-UHHD scoring systems in two studies.

**Table 1:** Overview of the datasets used across the four included studies, detailing the number of melanoma, nevus, and healthy cases; total patients and percentage of females; patient age distribution; tumor staging and thickness; presence of drusen; diagnostic methods; model architecture; and imaging modality. Diagnostic methods include MOLES (Management of Lesions Evaluation System), TFSOM-UHHD (Thickness, Fluid, Symptoms, Orange Pigment, Margin, Ultrasound Hollowness, Halo, Drusen), and confirmed clinical diagnoses from institutional databases

Study	Melanoma	Nevus	Healthy	Total patients (n% female)	Mean age (UM)	Mean age (Nevus)	Tumor stage (melanoma)	Tumor stage (Nevus)	Tumor thickness (mm)	Drusen	Diagnosis	Model used	Imaging modality	QUADAS-AI	F1-score	Accuracy
Jackson et al., 2024 [5]	2073 (18,510*)	1698 (8671*)	498 (1192*)	4255 (50.4%)	62 (19–99)	66 (18–96)	T1–626; T2–633; T3–703; T4–108	T1–1478; T2–202; T3–82; T4–0	UM: 4.0 (0.2–18.3); Nevus: 1.4 (0.1–9.9)	UM: 174; Nevus: 839	NA	RETFound	UWF images	Low risk	0.92	0.83
Sabazade et al., 2024 [21]	219 (29**; 17***)	583 (57**; 36***)	NA	802 (47.6%)	58 (14)	58 (14)	NA	NA	UM: > 2: 85; < 2: 129 (> 2: 17; < 2: 12**; > 2: 7; < 2: 10***); Nevus: > 2: 24; < 2: 432 (> 2: 2; < 2: 41**; > 2: 0; < 2: 36***)	NA	MOLES and TFSOM-UHHD	Custom model	UWF and standard images	Low risk	0.79**; 0.82***	NA
Hoffman et al., 2024 [22]	422	340	NA	723 (NA)	NA	NA	NA	NA	NA	NA	MOLES	ResNet 50	UWF and standard images	Low risk	0.92	0.91
Ganguly et al., 2019 [23]	110	60	NA	170 (NA)	NA	NA	NA	NA	NA	NA	NA	Custom model	Standard images	Low risk	0.95	0.92

*Indicates the total number of images; **Refers to the subset used for model development; ***Refers to the subset used for external validation

*UM* uveal melanoma, *UWF* ultra-widefield, *NA* not available

### Diagnostic accuracy analysis

**Fig. 2:**
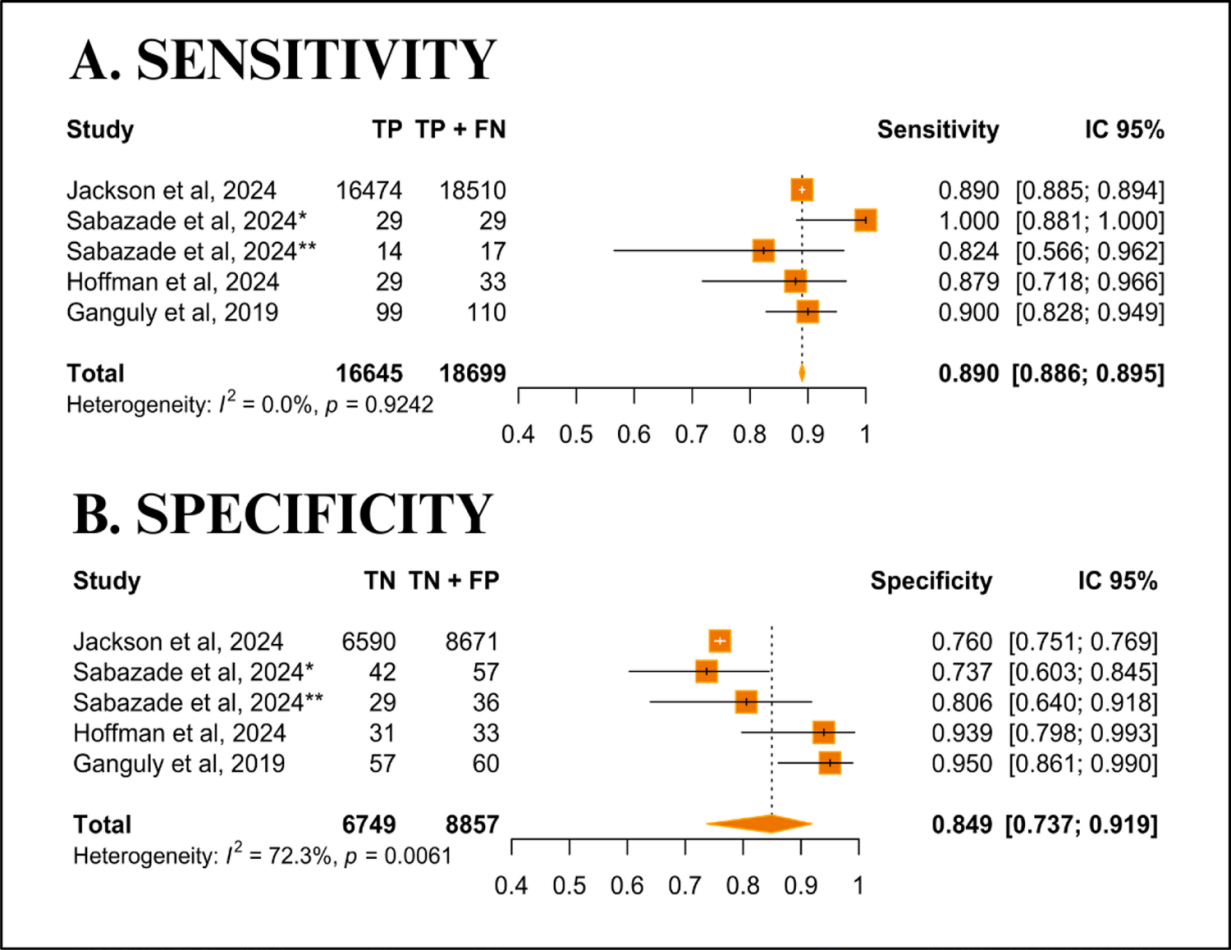
Sensitivity and specificity plots of deep learning-based detection models for uveal melanoma

The pooled sensitivity across the 5 included studies was 89.0% (95% CI 88.6–89.5%), with no significant heterogeneity (*I*^2^ = 0.0%, *p* = 0.9242), indicating a high level of consistency in the model’s ability to correctly identify uveal melanoma. Individual sensitivities ranged from 82.4 to 100.0%, with narrower confidence intervals in larger cohorts, particularly in Jackson et al. (2024), which contributed the greatest weight to the overall estimate. A total of 2054 false negatives were observed across studies (Fig. 2A).

The pooled specificity was 84.9% (95% CI 73.7–91.9%), but showed substantial heterogeneity (*I*^2^ = 72.3%, *p* = 0.0061), suggesting variability in the model’s ability to correctly identify nevi, indicating that not all detected lesions definitively correspond to melanoma. This comparative analysis was conducted utilizing various imaging modalities and a human-controlled reference group, factoring in variables such as clinical experience and expertise.

Specificity estimates ranged from 73.7 to 95.0%, with Jackson et al. (2024) reporting the lowest value at 76.0% (95% CI 75.1–76.9%) and Ganguly et al. (2019) reporting the highest at 95.0% (95% CI 86.1–99.0%), as shown in Fig. 2B.

**Fig. 3:**
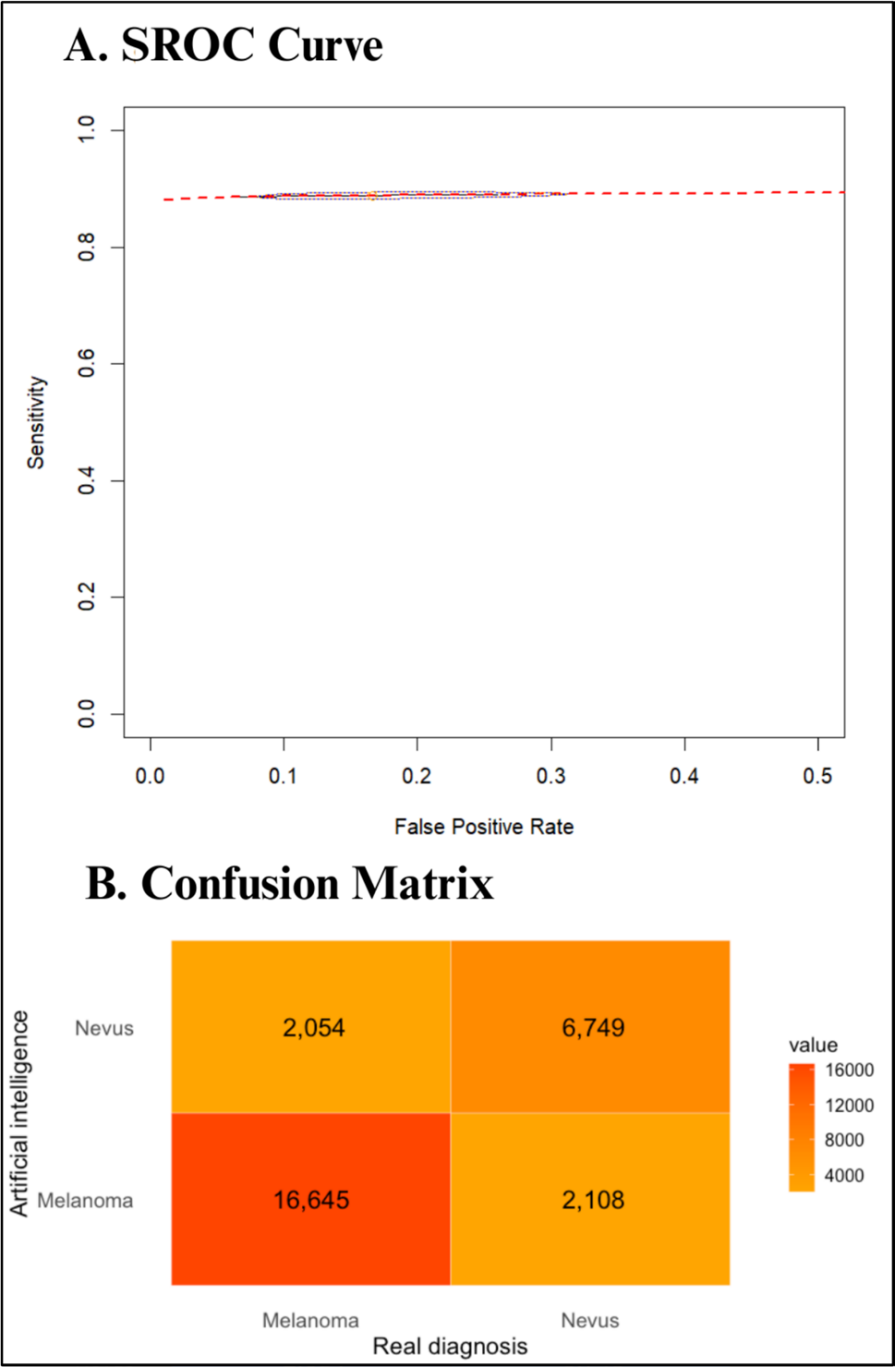
Summary Receiver Operating Characteristic (SROC) curve for deep learning-based detection of uveal melanoma (**A**). Aggregated confusion matrix combining all studies, comparing predicted diagnosis by the artificial intelligence model with the real diagnosis (**B**)

To assess the diagnostic performance of the deep learning models, a bivariate random-effects meta-analysis was conducted using the restricted maximum-likelihood (REML) estimation method. The resulting summary receiver-operating characteristic (SROC) curve demonstrated a pooled sensitivity of 0.889 (95% CI 0.884–0.893) and a pooled false positive rate of 0.166 (95% CI 0.093–0.278), as shown in Fig. 3A. The area under the curve (AUC) was 0.884, and the normalized partial AUC, restricted to the observed false positive rates, was 0.879—both indicating high overall diagnostic accuracy. Heterogeneity was minimal, with an *I*^2^ of 0.1% for sensitivity and ranging from 0.6 to 1.1% for specificity, depending on the adjustment method. The fitted SROC curve reflects consistent performance across studies, characterized by low variability in sensitivity and slightly greater dispersion in false positive rates.

**Fig. 4:**
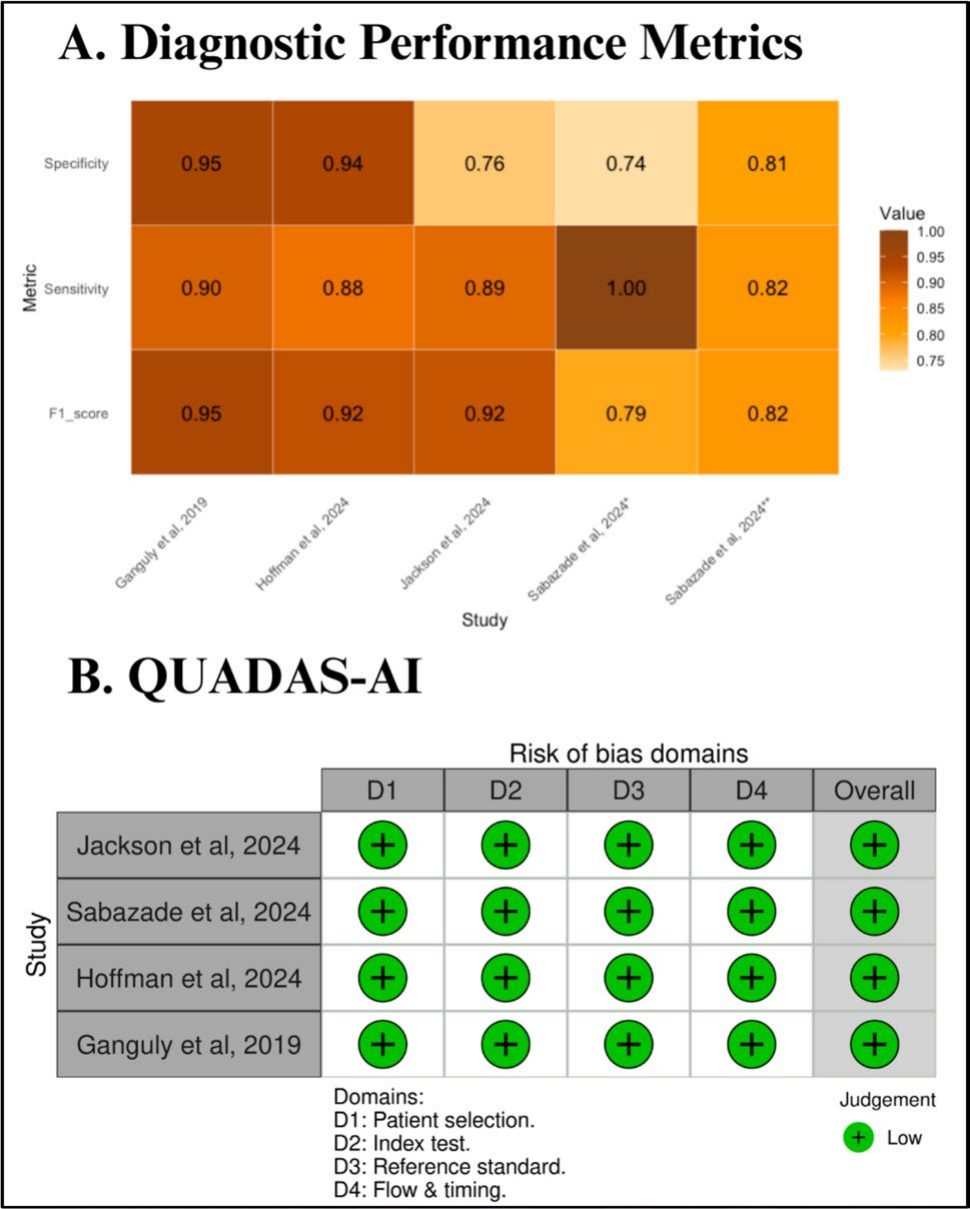
Summary of Diagnostic Performance and Risk of Bias Assessment. Heatmap showing the sensitivity, specificity, and F1-score values for each included study, calculated from confusion matrix components. Color intensity corresponds to the magnitude of each metric, with darker tones indicating higher performance (**A**). Risk of bias assessment using the QUADAS-AI tool. All studies showed low risk across all domains and overall judgment (**B**)

The confusion matrix shown in Fig. 3B summarizes the aggregated classification outcomes across all four studies, comprising 27,556 image samples. The model correctly identified 16,645 of the 18,699 true melanomas (sensitivity 89.0%) and 6749 of the 8857 nevi (specificity 76.2%). The positive predictive value (PPV) was 88.8%, and the negative predictive value (NPV) was 76.7%, resulting in an overall classification accuracy of 84.9%. While sensitivity remained consistent with the pooled estimate from the meta-analysis, the specificity was influenced by the larger cohort (Jackson et al.), in which specificity was comparatively lower. This suggests that, although the model performs reliably in detecting melanomas, there remains a moderate false-positive rate that may lead to unnecessary clinical follow-up in benign cases. Figure 4A provides a study-level breakdown of diagnostic performance metrics, including sensitivity, specificity, and F1-score for each individual study. The heatmap visually highlights the variation in performance across datasets, with darker shades indicating higher values.

The F1 score could not be formally pooled in the meta-analysis, because the included studies only reported the mean values, rather than the raw data necessary for aggregation. Therefore, these values are showed in Table 1 and Fig. 4A in the results section.

### Sensitive analysis

The Deeks’ funnel plot was visually inspected to assess potential publication bias among the included studies. Although the number of studies was limited, the plot exhibited noticeable asymmetry, suggesting a possible risk of publication bias. Due to the small sample size, no statistical test for funnel plot asymmetry was performed, and interpretation was restricted to visual assessment only. The Deeks’ plot can be visualized at Supplementary Fig. 1.

### Quality assessment

All included studies were judged to be at low risk of bias across every QUADAS-AI domain: patient selection, index test, reference standard, and flow and timing, owing to transparent reporting of eligibility criteria, prespecified thresholds for test positivity, use of independent validation cohorts, and complete inclusion of all enrolled cases. Consequently, the overall risk of bias across the body of evidence was deemed low. The QUADAS-AI evaluation of the included studies is summarized in Fig. 4B, with the full questionnaire detailed in Supplementary Table 5.

## Discussion

Our systematic review and diagnostic accuracy meta-analysis, which included 5 studies, evaluated the application of deep learning for the diagnosis of uveal melanoma based on images from 6388 patients aged between 58 and 63.2 years, of whom 2981 were diagnosed with uveal melanoma, 2563 with choroidal nevi, and 844 were healthy individuals. Our findings support that the application of artificial intelligence in the diagnosis of uveal melanoma may represent a promising tool in clinical practice, demonstrating a pooled sensitivity of 89.0% (95% CI 88.6–89.5%) and specificity of 84.9% (95% CI 73.7–91.9%). Similarly, the study by Zhang et al. [15] investigated the use of deep learning for monitoring neuroblastoma, the most common ocular cancer in childhood, and reported a sensitivity of 0.979 (95% CI 0.927–1.000) and a specificity of 1.000 (95% CI 1.000–1.000). These diagnostic performance results were not inferior to those achieved by ophthalmologists with 2–5 years of clinical experience.

In the absence of studies specifically evaluating deep learning for the identification of uveal melanoma, such algorithms have already been applied to other solid tumors, including lung, skin, and orbital tumors, demonstrating satisfactory performance in practical applications. The study by Faes et al. [24] analyzed a deep learning-based software designed for medical image classification by healthcare professionals without coding or deep learning expertise. The discriminative performance was assessed using the area under the precision–recall curve (AUPRC). The results supported the diagnostic capability of the tool, with significantly high discriminative performance after internal validation, particularly in tasks involving binary classification (sensitivity ranging from 73.3 to 97.0%; specificity from 67 to 100%; AUPRC from 0.87 to 1.00) [24]. Similarly, our meta-analysis showed an AUC of 0.88 for retinal image classification, indicating consistency between our findings and the existing literature.

Interestingly, the study by Faes et al.[24] also demonstrated that the diagnostic properties of deep learning models vary substantially when applied to tasks involving multiclass classification, with sensitivity ranging from 38 to 100% and specificity from 67 to 100%. This variability highlights the inherent heterogeneity of both the method and the specific algorithm employed—an important consideration when evaluating the feasibility of automating classification tasks in clinical practice. To date, three major challenges hinder the implementation of deep learning in healthcare. First, the development of a well-curated, classified, and labeled dataset in a computationally tractable format—ideally derived from clinically validated data—remains a significant hurdle [24, 25]. Second, the financial feasibility of implementation is a major concern. While deep learning models have undergone a revolution in terms of graphical processing capabilities, the field continues to evolve rapidly. Increasingly, companies are designing their own specialized hardware chips equipped with tensor processing units and field-programmable gate arrays, which require continuous financial adaptation in a fast-moving technological landscape [26, 27]. Third, the successful deployment of deep learning in healthcare requires professionals with a high level of technical and mathematical expertise, particularly in programming and model development. Although a report by bain & company indicates a substantial global increase in the number of AI specialists, this supply remains far from meeting the current demand [28].

Even though our findings point toward an optimistic future, an important concern regarding the application of deep learning lies in the degree of trust that physicians place in the clinical utility of this technology [29]. This is particularly relevant in cases of indeterminate choroidal tumors, where diagnosis based on histological findings obtained through biopsy (the gold standard) has a reported diagnostic accuracy of approximately 90% [30, 31]. This level of precision is likely to be surpassed by deep learning algorithms in the coming years, given the adaptive nature of these models. Deep learning systems can be continuously improved and updated with new examples without the need for complete retraining, especially when trained on detailed clinical datasets that include elastic image deformations, variations in color, and different imaging slices. Such diversity can enhance the robustness of training and, consequently, improve the diagnostic accuracy of these tools. Nevertheless, in the current stage of implementation, relying solely on deep learning algorithms over human clinical judgment—particularly in high-risk scenarios—demands a higher standard of accuracy and validation before integration into routine clinical practice. In the short term, however, artificial intelligence could serve as a valuable triage tool for optometrists and ophthalmologists, assisting in clinical decision-making processes that incorporate multimodal data, such as clinical history, imaging, genetic information, and biopsy results when required [32, 32].

Although our meta-analysis presents encouraging findings and suggests that the application of deep learning in the diagnosis of uveal melanoma may be promoted as a practical tool, it is essential to acknowledge some important limitations of this study. First, there is a lack of external validation for the imaging modalities investigated. While the reference standard was based on assessments by retinal specialists using multimodal imaging, the definitive diagnosis of the tumor can only be confirmed through histopathological analysis of choroidal biopsy specimens—a method associated with several potential complications, including tumor cell dissemination. As a result, choroidal biopsy is not routinely employed in clinical ophthalmic oncology. Therefore, the data included in our analysis may also encompass indeterminate lesions, the clinical management of which remains a topic of ongoing debate among specialists. Second, the variability in melanoma images derived from different imaging techniques and irradiation settings may contribute to the high heterogeneity observed in sensitivity estimates (*I*^2^ = 72.3%). Nonetheless, despite these limitations, our study still supports meaningful conclusions. Specifically, the application of deep learning—even as a relatively recent approach—should be encouraged in prospective clinical research, especially considering the high specificity observed, which was associated with no heterogeneity (*I*^2^ = 0). We recommend that future studies incorporate visual interpretability techniques such as gradient-weighted class activation mapping (Grad-CAM), which could enhance the transparency and interpretability of deep learning–based models. Grad-CAM highlights, within the analyzed images, the regions and visual patterns that most contributed to the model’s decision by generating heatmaps overlaid on the original ocular image [33, 34]. This allows clinicians to better understand the basis and rationale behind the model’s classification.

## Conclusions

Although fundus imaging is widely available in routine outpatient settings and in the evaluation of ophthalmologic disorders, multimodal imaging is generally restricted to specialized clinics. Our review and meta-analysis strongly supports deep learning as a highly promising and potentially valuable tool for the differential diagnosis of uveal melanoma in clinical practice. The combined results indicated a high diagnostic performance, effectively discriminating between patients with uveal melanoma and individuals with other conditions, such as choroidal nevi, or healthy status. Crucially, the achieved diagnostic performance metrics were found to be noninferior to the accuracy rates attained by ophthalmologists possessing an intermediate level of clinical experience. Therefore, the development of softwares capable of analyzing fundus photographs could enable prestratification, potentially improving the early diagnosis of uveal melanoma and representing a cost-effective alternative.

## Supplementary Information

Below is the link to the electronic supplementary material.

Supplementary file1 (DOCX 2028 KB)

## Data Availability

The datasets generated and/or analyzed during the current study are available within the manuscript or supplementary information files.
