# Supplementary material for: Deep learning for early diagnosis of uveal melanoma: a systematic review and meta-analysis
Source: Clin Transl Oncol. 2026 Jan 30;28(7):2854–63. doi: 10.1007/s12094-025-04187-3 (PMC13282208; doi:10.1007/s12094-025-04187-3)

**Supplementary Material**

**Supplementary Table 1** PRISMA 2020 Checklist.

**Supplementary Table 2** PRISMA 2020 for Abstract Checklist.

**Supplementary Table 3** Inclusion and exclusion criteria of included studies

**Supplementary Table 4** Search strategies.

**Supplementary Figure 1.** Deeks Funnel Plot

**Table S1. PRISMA 2020 Checklist.**

| Section and Topic | Item # | Checklist item | | Location where item is reported |
| --- | --- | --- | --- | --- |
| TITLE | | | |  |
| Title | 1 | Identify the report as a systematic review. | | Page 1 |
| ABSTRACT | | | |  |
| Abstract | 2 | See the PRISMA 2020 for Abstracts checklist. | | Supplementary Materials: Table S3. |
| INTRODUCTION | | | |  |
| Rationale | 3 | Describe the rationale for the review in the context of existing knowledge. | | Page 1 |
| Objectives | 4 | Provide an explicit statement of the objective(s) or question(s) the review addresses. | | Page 2 |
| METHODS | | | |  |
| Eligibility criteria | 5 | Specify the inclusion and exclusion criteria for the review and how studies were grouped for the syntheses. | | Pages 2-3 |
| Information sources | 6 | Specify all databases, registers, websites, organisations, reference lists and other sources searched or consulted to identify studies. Specify the date when each source was last searched or consulted. | | Page 3 |
| Search strategy | 7 | Present the full search strategies for all databases, registers and websites, including any filters and limits used. | | Supplementary Materials: Table S4 Search Strategies |
| Selection process | 8 | Specify the methods used to decide whether a study met the inclusion criteria of the review, including how many reviewers screened each record and each report retrieved, whether they worked independently, and if applicable, details of automation tools used in the process. | | Page 3 |
| Data collection process | 9 | Specify the methods used to collect data from reports, including how many reviewers collected data from each report, whether they worked independently, any processes for obtaining or confirming data from study investigators, and if applicable, details of automation tools used in the process. | | Page 3 |
| Data items | 10a | List and define all outcomes for which data were sought. Specify whether all results that were compatible with each outcome domain in each study were sought (e.g. for all measures, time points, analyses), and if not, the methods used to decide which results to collect. | | Pages 3 |
|  | 10b | List and define all other variables for which data were sought (e.g. participant and intervention characteristics, funding sources). Describe any assumptions made about any missing or unclear information. | | Page NO |
| Study risk of bias assessment | 11 | Specify the methods used to assess risk of bias in the included studies, including details of the tool(s) used, how many reviewers assessed each study and whether they worked independently, and if applicable, details of automation tools used in the process. | | Page 4 |
| Effect measures | 12 | Specify for each outcome the effect measure(s) (e.g. risk ratio, mean difference) used in the synthesis or presentation of results. | | Page  4 |
| Synthesis methods | 13a | Describe the processes used to decide which studies were eligible for each synthesis (e.g. tabulating the study intervention characteristics and comparing against the planned groups for each synthesis (item #5)). | | Pages 2 and 3 |
|  | 13b | Describe any methods required to prepare the data for presentation or synthesis, such as handling of missing summary statistics, or data conversions. | | Pages 4 |
|  | 13c | Describe any methods used to tabulate or visually display results of individual studies and syntheses. | | Page 3 |
|  | 13d | Describe any methods used to synthesize results and provide a rationale for the choice(s). If meta-analysis was performed, describe the model(s), method(s) to identify the presence and extent of statistical heterogeneity, and software package(s) used. | | Page 4 |
|  | 13e | Describe any methods used to explore possible causes of heterogeneity among study results (e.g. subgroup analysis, meta-regression). | | Pages 4 |
|  | 13f | Describe any sensitivity analyses conducted to assess robustness of the synthesized results. | | Pages 4 |
| Reporting bias assessment | 14 | Describe any methods used to assess risk of bias due to missing results in a synthesis (arising from reporting biases). | | Pages 4 |
| Certainty assessment | 15 | Describe any methods used to assess certainty (or confidence) in the body of evidence for an outcome. | | Pages 4 |
| RESULTS | | | |  |
| Study selection | 16a | Describe the results of the search and selection process, from the number of records identified in the search to the number of studies included in the review, ideally using a flow diagram. | | Page 4-5  Figure 1 |
|  | 16b | Cite studies that might appear to meet the inclusion criteria, but which were excluded, and explain why they were excluded. | Page NO | |
| Study characteristics | 17 | Cite each included study and present its characteristics. | | Pages 5-6  Table 1 |
| Risk of bias in studies | 18 | Present assessments of risk of bias for each included study. | | Supplementary Materials: Figure 4. |
| Results of individual studies | 19 | For all outcomes, present, for each study: (a) summary statistics for each group (where appropriate) and (b) an effect estimate and its precision (e.g. confidence/credible interval), ideally using structured tables or plots. | | Pages 7-9  Figure 2 and 3 |
| Results of syntheses | 20a | For each synthesis, briefly summarise the characteristics and risk of bias among contributing studies. | | Pages 7-9  Supplementary Materials: Figure 4 |
|  | 20b | Present results of all statistical syntheses conducted. If meta-analysis was done, present for each the summary estimate and its precision (e.g. confidence/credible interval) and measures of statistical heterogeneity. If comparing groups, describe the direction of the effect. | | Pages 7-9  Figure 2 and 3  Supplementary Materials: Figure 1, 2 and 3 |
|  | 20c | Present results of all investigations of possible causes of heterogeneity among study results. | | Page 9  Figure 4 |
|  | 20d | Present results of all sensitivity analyses conducted to assess the robustness of the synthesized results. | | Page 8-9  Supplementary Materials: Figure 2 |
| Reporting biases | 21 | Present assessments of risk of bias due to missing results (arising from reporting biases) for each synthesis assessed. | | Page NO |
| Certainty of evidence | 22 | Present assessments of certainty (or confidence) in the body of evidence for each outcome assessed. | | Page 9 |
| DISCUSSION | | | |  |
| Discussion | 23a | Provide a general interpretation of the results in the context of other evidence. | | Pages 9-11 |
|  | 23b | Discuss any limitations of the evidence included in the review. | | Page 10-11 |
|  | 23c | Discuss any limitations of the review processes used. | | Page 10-11 |
|  | 23d | Discuss implications of the results for practice, policy, and future research. | | Page 10 |
| OTHER INFORMATION | | | |  |
| Registration and protocol | 24a | Provide registration information for the review, including register name and registration number, or state that the review was not registered. | | Page 2 |
|  | 24b | Indicate where the review protocol can be accessed, or state that a protocol was not prepared. | | Page 2 |
|  | 24c | Describe and explain any amendments to information provided at registration or in the protocol. | | Page 2 |
| Support | 25 | Describe sources of financial or non-financial support for the review, and the role of the funders or sponsors in the review. | | Page 12 |
| Competing interests | 26 | Declare any competing interests of review authors. | | Page 12 |
| Availability of data, code and other materials | 27 | Report which of the following are publicly available and where they can be found: template data collection forms; data extracted from included studies; data used for all analyses; analytic code; any other materials used in the review. | | Page 12 |

**Table S2. PRISMA 2020 for Abstract Checklist.**

| Section and Topic | Item # | Checklist item | Reported (Yes/No) |
| --- | --- | --- | --- |
| TITLE | | |  |
| Title | 1 | Identify the report as a systematic review. | Yes |
| BACKGROUND | | |  |
| Objectives | 2 | Provide an explicit statement of the main objective(s) or question(s) the review addresses. | Yes |
| METHODS | | |  |
| Eligibility criteria | 3 | Specify the inclusion and exclusion criteria for the review. | No |
| Information sources | 4 | Specify the information sources (e.g. databases, registers) used to identify studies and the date when each was last searched. | Yes |
| Risk of bias | 5 | Specify the methods used to assess risk of bias in the included studies. | No |
| Synthesis of results | 6 | Specify the methods used to present and synthesise results. | No |
| RESULTS | | |  |
| Included studies | 7 | Give the total number of included studies and participants and summarise relevant characteristics of studies. | Yes |
| Synthesis of results | 8 | Present results for main outcomes, preferably indicating the number of included studies and participants for each. If meta-analysis was done, report the summary estimate and confidence/credible interval. If comparing groups, indicate the direction of the effect (i.e. which group is favoured). | Yes |
| DISCUSSION | | |  |
| Limitations of evidence | 9 | Provide a brief summary of the limitations of the evidence included in the review (e.g. study risk of bias, inconsistency and imprecision). | No |
| Interpretation | 10 | Provide a general interpretation of the results and important implications. | Yes |
| OTHER | | |  |
| Funding | 11 | Specify the primary source of funding for the review. | No |
| Registration | 12 | Provide the register name and registration number. | No |

**Table S3** Inclusion and exclusion criteria of included studies

| **Study** | **Inclusion Criteria** | **Exclusion Criteria** |
| --- | --- | --- |
| DADZIE, 2024 | 1. Patients diagnosed with melanocytic choroidal tumours 2. Patients seen and clinically diagnosed at the University of Illinois at Chicago eye clinic between January 2010 and July 2023. 3. Ultra-widefield retinal images obtained from both eyes:  - Tumour eye (UM or choroidal naevus) - Fellow eye (used as control) | 1. Patients who had been treated prior to presentation 2. Fellow eyes with opaque ocular media 3. Fellow eyes with choroidal tumour |
| GANGULY, 2019 | 1. 170 pre-diagnosed ocular images 2. Images taken from the New York Eye Cancer Center database 3. Each image was assessed and verified by medical experts 4. Images labeled as either melanoma or non-melanoma 5. Images of various original resolutions 6. All images rescaled to 200×200 pixels for uniform input | 1. Images not verified by medical experts likely excluded 2. Images not fitting quality/resolution standards would be unsuitable for Convolutional Neural Network input |
| HOFFMAN, 2024 | 1. Subjects diagnosed with choroidal nevus, treatment-naïve choroidal melanoma, and irradiated choroidal melanoma, with irradiation involving either proton beam therapy or plaque radiotherapy. 2. Fundus photographs collected between January 2010 and January 2023 3. Images acquired using Optos (Daytona) and Clarus (ZEISS) fundus cameras 4. Diagnosis based on multimodal imaging by retinal specialists | 1. Inadequate imaging quality 2. Prior treatment at another clinic 3. Prior endoresection surgery |
| JACKSON, 2024 | 1. Fundus images obtained from 3942 patients at the Liverpool Ocular Oncology Centre 2. Images acquired between 1995 and 2020 3. Images captured using Optos ultrawidefield model P200 camera 4. Included image were:  - Good-quality - Classified as choroidal melanoma or nevus based on clinical diagnosis  1. The diagnosis was based on the known clinical features pertaining to tumor dimensions, orange pigment, subretinal fluid, and ultrasound features of reflectivity and Doppler phenomenon. 2. The diagnosis of the UM and nevus patients was undertaken by 2 experienced ocular oncologists involving direct patient examination, Optos and autofluorescence imaging, OCT scans, and ultrasonography. | 1. Images were excluded if (1) they were blurry or of low resolution; (2) they had the presence of artifacts, such as eyelashes protruding across the whole image; or (3) most of the eye was blocked by camera distortion. 2. Other excluding factors included the following: eyes with vitreous hemorrhage; a lesion that was over 50% out of view; dense cataract; no visible vascular structures; and any nevus that had subsequently transformed into a melanoma. |
| SABAZADE, 2024 | 1. Photograph taken after January 1, 2010, marking a period in which medical records were digitalized which facilitated control over follow-up. 2. Diagnosis of either choroidal melanoma (International Classification of Diseases, 10th revision C69.3) or choroidal nevi (International Classification of Diseases, 10th revision D31.3). 3. Diagnoses had to be established by a subspecialized ocular oncologist. 4. For lesions diagnosed as nevi at the time of photography, there had to be at least 5 years of follow-up without rediagnosis as a melanoma. 5. Lesions that were diagnosed melanoma at a later point in time (e.g., due to growth) were considered melanomas in this study. | 1. Photographs of low quality, where issues such as focus, movement artifacts, overexposure or underexposure, and reflections hindered the determination of lesion extent or the presence of features like orange pigment or drusen. 2. Photographs where our assessment determined that less than half of the lesion was visible. 3. Lesion obscured by retinal detachment, vitreous bleeding or similar. |

**Table S4** Search Strategies

| **Database** | **Search Strategy** |
| --- | --- |
| **PubMed** | ("Uveal Neoplasms" OR "Uveal Melanoma" OR "Intraocular Melanoma" OR "Melanoma, Intraocular" OR "Melanoma Of The Uvea" OR "Melanoma, Uveal" OR "Ocular Melanoma" OR "Melanoma, Ocular" OR "Choroidal Melanoma" OR "Choroidal Melanomas" OR "Melanoma, Choroidal" OR "Iris Melanoma" OR "Melanoma, Iris" OR "Melanomas, Iris" OR "Ciliary Body Melanoma" OR "Body Melanoma, Ciliary" OR "Ciliary Body Melanomas" OR "Melanoma, Ciliary Body" OR "Ciliochoroidal Melanoma" OR "Melanoma, Ciliochoroidal" OR "Eye Melanoma") AND ("Artificial Intelligence" OR "Machine Learning" OR "Deep Learning" OR "Deep Learning Algorithm" OR "Neural Network" OR "AI" OR "Computer-Aided Diagnosis" OR "Automated Diagnosis" OR "Algorithm" OR "Intelligence, Artificial" OR "Computer Reasoning" OR "Reasoning, Computer" OR "AI (Artificial Intelligence)" OR "Machine Intelligence" OR "Intelligence, Machine" OR "Computational Intelligence" OR "Intelligence, Computational" OR "Computer Vision Systems" OR "Computer Vision System" OR "System, Computer Vision" OR "Systems, Computer Vision" OR "Vision System, Computer" OR "Vision Systems, Computer") |
| **Scopus** | ((TITLE-ABS-KEY("Uveal Neoplasms") OR TITLE-ABS-KEY("Uveal Melanoma") OR TITLE-ABS-KEY("Intraocular Melanoma") OR TITLE-ABS-KEY("Melanoma, Intraocular") OR TITLE-ABS-KEY("Melanoma Of The Uvea") OR TITLE-ABS-KEY("Melanoma, Uveal") OR TITLE-ABS-KEY("Ocular Melanoma") OR TITLE-ABS-KEY("Melanoma, Ocular") OR TITLE-ABS-KEY("Choroidal Melanoma") OR TITLE-ABS-KEY("Choroidal Melanomas") OR TITLE-ABS-KEY("Melanoma, Choroidal") OR TITLE-ABS-KEY("Iris Melanoma") OR TITLE-ABS-KEY("Melanoma, Iris") OR TITLE-ABS-KEY("Melanomas, Iris") OR TITLE-ABS-KEY("Ciliary Body Melanoma") OR TITLE-ABS-KEY("Body Melanoma, Ciliary") OR TITLE-ABS-KEY("Ciliary Body Melanomas") OR TITLE-ABS-KEY("Melanoma, Ciliary Body") OR TITLE-ABS-KEY("Ciliochoroidal Melanoma") OR TITLE-ABS-KEY("Melanoma, Ciliochoroidal") OR TITLE-ABS-KEY("Eye melanoma")) AND (TITLE-ABS-KEY("Artificial Intelligence") OR TITLE-ABS-KEY("machine learning") OR TITLE-ABS-KEY("deep learning") OR TITLE-ABS-KEY("deep learning algorithm") OR TITLE-ABS-KEY("neural network") OR TITLE-ABS-KEY("AI") OR TITLE-ABS-KEY("computer-aided diagnosis") OR TITLE-ABS-KEY("automated diagnosis") OR TITLE-ABS-KEY("algorithm") OR TITLE-ABS-KEY("Intelligence, Artificial") OR TITLE-ABS-KEY("Computer Reasoning") OR TITLE-ABS-KEY("Reasoning, Computer") OR TITLE-ABS-KEY("AI (Artificial Intelligence)") OR TITLE-ABS-KEY("Machine Intelligence") OR TITLE-ABS-KEY("Intelligence, Machine") OR TITLE-ABS-KEY("Computational Intelligence") OR TITLE-ABS-KEY("Intelligence, Computational") OR TITLE-ABS-KEY("Computer Vision Systems") OR TITLE-ABS-KEY("Computer Vision System") OR TITLE-ABS-KEY("System, Computer Vision") OR TITLE-ABS-KEY("Systems, Computer Vision") OR TITLE-ABS-KEY("Vision System, Computer") OR TITLE-ABS-KEY("Vision Systems, Computer")) |
| **Web of Science** | TS=("Uveal Neoplasms" OR TS="Uveal Melanoma" OR TS="Intraocular Melanoma" OR TS="Melanoma, Intraocular" OR TS="Melanoma Of The Uvea" OR TS="Melanoma, Uveal" OR TS="Ocular Melanoma" OR TS="Melanoma, Ocular" OR TS="Choroidal Melanoma" OR TS="Choroidal Melanomas" OR TS="Melanoma, Choroidal" OR TS="Iris Melanoma" OR TS="Melanoma, Iris" OR TS="Melanomas, Iris" OR TS="Ciliary Body Melanoma" OR TS="Body Melanoma, Ciliary" OR TS="Ciliary Body Melanomas" OR TS="Melanoma, Ciliary Body" OR TS="Ciliochoroidal Melanoma" OR TS="Melanoma, Ciliochoroidal" OR TS="Eye Melanoma") AND TS=("Artificial Intelligence" OR TS="Machine Learning" OR TS="Deep Learning" OR TS="Deep Learning Algorithm" OR TS="Neural Network" OR TS="AI" OR TS="Computer-Aided Diagnosis" OR TS="Automated Diagnosis" OR TS="Algorithm" OR TS="Intelligence, Artificial" OR TS="Computer Reasoning" OR TS="Reasoning, Computer" OR TS="AI (Artificial Intelligence)" OR TS="Machine Intelligence" OR TS="Intelligence, Machine" OR TS="Computational Intelligence" OR TS="Intelligence, Computational" OR TS="Computer Vision Systems" OR TS="Computer Vision System" OR TS="System, Computer Vision" OR TS="Systems, Computer Vision" OR TS="Vision System, Computer" OR TS="Vision Systems, Computer") |

**Supplementary Figure 1.** Deeks Funnel Plot.


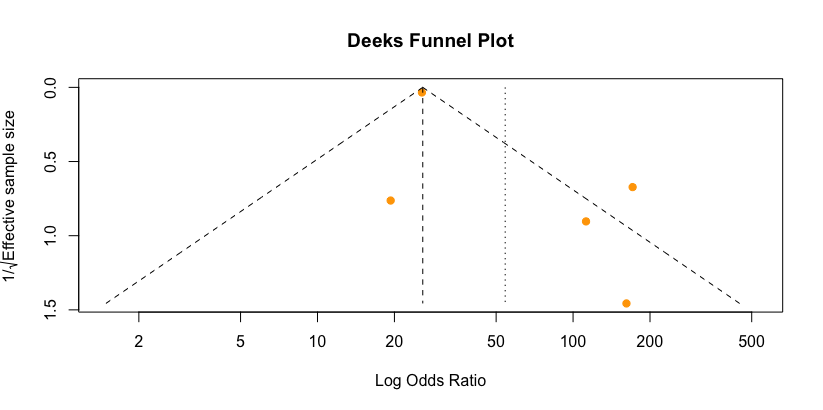

Supplement: Supplementary file 1 — Supplementary file1 (DOCX 2028 KB) [file 12094_2025_4187_MOESM1_ESM.docx]
